# Store-and-Forward Teledermatology for Assessing Skin Cancer in 2023: Literature Review

**DOI:** 10.2196/43395

**Published:** 2023-05-17

**Authors:** Leah Kirsten Jones, Amanda Oakley

**Affiliations:** 1 Te Whatu Ora Waikato Hamilton New Zealand; 2 The University of Auckland Auckland New Zealand

**Keywords:** telemedicine, skin neoplasm, skin cancer, store-and-forward, melanoma, squamous cell carcinoma, basal cell carcinoma, cancer, dermoscopy, mobile phone

## Abstract

**Background:**

The role of teledermatology for skin lesion assessment has been a recent development, particularly, since the COVID-19 pandemic has impacted the ability to assess patients in person. The growing number of studies relating to this area reflects the evolving interest.

**Objective:**

This literature review aims to analyze the available research on store-and-forward teledermatology for skin lesion assessment.

**Methods:**

MEDLINE was searched for papers from January 2010 to November 2021. Papers were searched for assessment of time management, effectiveness, and image quality.

**Results:**

The reported effectiveness of store-and-forward teledermatology for skin lesion assessment produces heterogeneous results likely due to significant procedure variations. Most studies show high accuracy and diagnostic concordance of teledermatology compared to in-person dermatologist assessment and histopathology. This is improved through the use of teledermoscopy. Most literature shows that teledermatology reduces time to advice and definitive treatment compared to outpatient clinic assessment.

**Conclusions:**

Overall, teledermatology offers a comparable standard of effectiveness to in-person assessment. It can save significant time in expediting advice and management. Image quality and inclusion of dermoscopy have a considerable bearing on the overall effectiveness.

## Introduction

### Background

The number of studies relating to teledermatology continues to rise exponentially. The reasons for this are multitude, including the COVID-19 pandemic limiting in-person consults, advances in technology meaning better availability of telemedicine and, in particular, teledermoscopy, as well as more familiarity and interest from health care professionals in using teledermatology. There are various methods of teledermatology. The main distinguishing factor is whether a pathway uses video consultations or store-and-forward, also known as advice and guidance. The former uses real-time technology to provide assessment, while the latter involves taking images for later review. Teledermatology can also be distinguished by the specialties involved, whether this is the more common general practitioner (GP) to a dermatologist, dermatologist to dermatologist, GP to GP, or patient to GP.

There are many variables to consider when assessing store-and-forward teledermatology due to the variation in the way it is implemented and assessed. This review aims to collate information from the breadth of available data.

### Objective

This literature review discusses time to advice, effectiveness, and image quality for store-and-forward teledermatology for skin lesion assessment.

## Methods

MEDLINE was searched using the keywords “telemedicine” or “teledermatology” and “skin neoplasms” or “skin cancer” for papers from January 2010 to November 2021. The period was chosen due to the inclusion of several review papers that summarize prominent earlier work as well as significant advances in technology over the last 10 years. A single database search was chosen to maximize efficiency, reliability, and reproducibility. A narrative review method was selected to allow flexibility in discussing the heterogeneous results for each outcome of interest. Papers about store-and-forward teledermatology for skin lesion assessment reporting effects on time, effectiveness, and image quality were included. All study designs were included, including reviews, interventional studies, and observational studies.

## Results

In total, 45 papers meeting the inclusion criteria were identified after removing duplicate reports, of which, 4 were review papers, 10 were interventional studies (including 2 randomized controlled trials), and 31 were observational studies.

### Time to Advice and Management

In total, 11 papers reporting time outcomes were identified with measures including time to advice, time to biopsy, and time to definitive surgery. The results are outlined in Table 1.

**Table 1:** Studies reporting time to advice and management.

Type	Setting	Sample	Outcome	Reference
Observational	Single-center study in the United States	212 patients (146 teledermatology consults and 66 in-person consults)	Decreased time to treatment by 2 weeks. Increased the percentage of lesions treated within 60 days.	Lee et al [1]
Observational	Single-center study in Australia	Aggregated probabilities analysis	Mean time to clinical resolution was 9 (range 1-50) days with teledermoscopy referral compared with 35 (range 0-138 days) days for conventional referrals.	Snoswell et al [2]
Observational	Single-center study in New Zealand	613 lesions in 310 patients	Median time between referral and attendance at the virtual clinic was 9 days compared to 26.5 days for conventional referrals.	Congalton et al [3]
Observational	Single-center study in the United States	293 patients	Mean time to biopsy of skin cancer was 9.7 (median 9.0) days for teledermatology referrals compared to 13.8 days for conventional referrals (median 12.0 days).	Kahn et al [4]
Observational	Single-center study in the United States	79 referrals	Median time to evaluation was 0.5 (IQR 0.172-0.94) days for teledermatology referrals compared to 70.0 (IQR 33.25-83.0) days for conventional referrals.	Carter et al [5]
Observational	Single-center study in the United States	2385 referrals (1258 conventional referrals and 1127 teledermatology referrals)	Implementation of teledermatology allowed median wait times to reduce from 77 to 28 days.	Naka et al [6]
Observational	Single-center study in Spain	43,677 patients	Average time to skin lesion advice of 72 hours for teledermatology referrals.	Moreno-Ramírez and Ferrándiz [7]
Observational	Single-center study in France	1079 referrals (1043 conventional referrals and 36 teledermatology referrals)	Teledermatology referrals resulted in a mean of 7.8 more days waiting for surgery compared to conventional referrals.	Duong et al [8]
Observational	Single-center study in the United States	1021 referrals (434 teledermatology referrals compared to 587 conventional referrals)	No significant difference found in time from initial consult to biopsy of suspicious lesions (47.3 vs 45.5 days; *P*=.8) or in time from biopsy to definitive treatment of malignant tumors (65.4 vs 67.5 days; *P*=.8) for teledermatology referrals compared to conventional referrals.	Creighton-Smith et al [9]
Observational	Single-center study in the United Kingdom	4589 teledermatology referrals	Teledermatology allowed a reduction of 86.3% (range 78%-93%) of the number of patients requiring in-person assessment.	Lowe et al [10]
Systematic review	N/A^a^	21 studies	Three studies reported reduced waiting times with teledermatology referrals.	Finnane et al [11]

^a^N/A: not applicable.

### Effectiveness

In total, 34 papers assessing the effectiveness of teledermatology assessment were identified. These included accuracy and its derivations, concordance, positive and negative predictive values, and impact on subsequent in-person assessment. The results are outlined in Table 2.

**Table 2:** Studies reporting the effectiveness of teledermatology.

Type	Setting	Sample	Outcome	Reference
Systematic review	N/A^a^	22 studies	Overall sensitivity 94.9% (95% CI 90-97.4) and specificity 84.3%.	Chuchu et al [12]
Observational	Single-center study in Austria	955 lesions	Diagnostic accuracy was 94% with sensitivity of 100% and specificity of 95.8%.	Massone et al [13]
Observational	Single-center study in Serbia	120 patients and 121 pigmented lesions	Diagnostic accuracy between teledermoscopy and histopathology was 90.91%.	Bandic et al [14]
Observational	Single-center study in Ukraine	314 lesions	Accuracy of 90.3%-100.0% for teledermatology assessment compared to in-person and 85.1%-8.9% compared to histopathological diagnoses.	Kravets et al [15]
Observational	Single-center study in the United States	7960 patients	45 (74%) of melanomas were correctly diagnosed, and 57 (93%) were correctly managed, resulting in similar diagnostic and management accuracy of teledermatology compared to in-person assessment.	Wang et al [16]
Observational	Single-center study in Brazil	39 patients	Comparable sensitivities of teledermatology in comparison to in-person assessment (80.8% for teledermatology compared to 80.8% for in-person).	Silveira et al [17]
Observational	Single-center study in Denmark	519 patients	No significant difference in sensitivity between teledermatology and conventional referrals. Specificity was lower in teledermatology referrals.	Vestergaard et al [18]
Observational	Single-center study in Spain	636 patients and 1000 keratotic lesions	The sensitivity, specificity, and positive and negative predictive values for actinic keratosis diagnosis by teledermatology were high (range 82.2-95.0).	Sola-Ortigosa et al [19]
Observational	Single-center study in Austria	113 lesions	High concordance, sensitivity, and specificity for all diagnostic categories.	Kroemer et al [20]
Interventional	Single-center study in New Zealand	200 patients and 491 lesions	Sensitivity of teledermoscopy assessment was close to 100%, and specificity was 90%.	Tan et al [21]
Observational	Single-center study in the United States	321 lesions	The sensitivity for keratinocytic skin cancer diagnosed by teledermatology was 92%, and specificity 49%, resulting in positive and negative predictive values of 61% and 88%.	Cotes et al [22]
Observational	Single-center study in the Netherlands	108 teledermatoscopy referrals	Agreement between teledermatologist and in-person assessment was κ=0.61 (substantial agreement) for diagnosis and κ=0.23 (fair) for management.	van der Heijden et al [23]
Systematic review	N/A	21 studies	Diagnostic accuracy for teledermatology assessment slightly inferior to in-person assessment at 51%-85% (κ=0.41-0.63) compared to 67%-85% (κ=0.90).	Finnane et al [11]
Observational	Single-center study in New Zealand	3470 referrals	Teledermatology assessment of pigmented lesions yielded a positive predictive value of 38.1% and number needed to excise of 2.6.	Sunderland et al [24]
Observational	Single-center study in the United States	8706 patients	69 lesions diagnosed as melanoma resulting in a positive predictive value of 13.7%.	Gemelas et al [25]
Observational	Single-center study in the United States	3021 lesions and 2152 patients	Agreement was fair to substantial for primary diagnosis (45.7%-80.1%; κ=0.32-0.62), substantial to almost perfect for aggregated diagnoses (primary plus differential; 78.6%-93.9%; κ=0.77-0.90), and fair for management (66.7%-86.1%; κ=0.28-0.41).	Warshaw et al [26]
Observational	Single-center study in the United States	1021 referrals (434 teledermatology referrals compared to 587 conventional referrals)	Perfect diagnostic concordance was 36% (18/50) for teledermatology consults compared to 43.1% (69/160) for in-person assessment (*P*=.4). Partial concordance (benign vs malignant) was higher for teledermatology consults (26/50, 52%) compared to in-person (58/160, 36.3%; *P*<.05).	Creighton-Smith et al [9]
Interventional	Single-center study in Sweden	172 lesions	No difference in agreement between teledermatology diagnoses between 2 image sets.	Dahlén Gyllencreutz et al [27]
Observational	Single-center study in the United States	79 referrals	Diagnostic concordance between teledermatologists was at least partially concordant in 79 (100%) patients. For those subsequently seen in-person at least partial concordance with teledermatologist was observed in 16/29 (89.7%).	Carter et al [5]
Interventional	Single-center study in Germany	26 patients	The concordance between teledermatologist and in-person assessment was 92.3% for diagnosis and 76.9% for management.	Zink et al [28]
Interventional	Single-center study in Austria	23 lesions	Agreement as calculated by prevalence and bias-adjusted κ showed almost perfect agreement (0.9-0.982).	Arzberger et al [29]
Interventional	Single-center study in the United States	137 lesions and 86 patients	Diagnostic concordance was 82% between the in-person dermatologist and the teledermatologist (95% CI 0.73-0.89), with a κ coefficient of 0.62 (good agreement).	Lamel et al [30]
Interventional	Single-center study in Italy	10 lesions	Teledermatology consults resulted in lower diagnostic concordance compared to in-person assessment and did not improve with the addition of teledermoscopy.	de Giorgi et al [31]
Observational	Single-center study in New Zealand	2108 lesions	1303 (83%) of 1571 lesions with histology available were found to be benign, and 260 (17%) lesions were diagnosed as melanoma, resulting in a melanoma-specific benign:malignant ratio of 5.0:1.	Greenwald et al [32]
Observational	Single-center study in the United States	59,279 patients	One teledermatology pathway resulted in 39% fewer in-person assessments (relative risk 0.61, 95% CI 0.57-0.65).	Marwaha et al [33]
Observational	Single-center study in the United Kingdom	40,201 teledermatology consultations, 77% lesions	Teledermatology allowed 50% of referrals to be discharged to general practice, and 33% to proceed straight to biopsy.	Mehrtens et al [34]
Observational	Single-center study in the United Kingdom	76 patients	Benign diagnoses were made in 52 (68%) patients avoiding subsequent in-person assessment.	Cheung et al [35]
Randomized controlled trial	Single-center study in Switzerland	981 lesions from 39 general practitioners	3 (1.5%) lesions triaged as requiring no further investigation were found to be malignant.	Tandjung et al [36]
Narrative review	N/A	5 papers included in the skin cancer surveillance discussion	Most guidelines suggest in-person review for suspected malignant lesions. Improved accuracy noted with teledermoscopy.	Beer et al [37]
Systematic review	N/A	6 studies	All studies concluded that “high-quality” and dermoscopy images improve diagnostic accuracy. None considered it an adequate replacement for in-person assessment.	Woodley [38]
Observational	Two-center study in Spain	395 consultations	Increased interobserver concordance found with the use of teledermoscopy, resulting in an increased coefficient of agreement from 0.486 to 0.641.	Gómez Arias et al [39]
Interventional	Single-center study in Turkey	150 patients	Diagnostic reliability (κ) for teledermatology without dermatoscopy was 0.75 and 0.77 for 2 different dermatologists, which increased with the addition of dermoscopy to 0.86 and 0.88 (*P*<.05).	Şenel et al [40]
Observational	Single-center study in Sweden	686 patients and 883 pigmented lesions	The sensitivity for the diagnosis of melanoma by means of teledermatology monitoring was 88.9%, and specificity 93.9%.	Berglund et al [41]
Observational	Single-center study in Sweden	157 referrals (80 teledermoscopy referrals and 77 conventional referrals)	The interobserver concordance was moderate with both teledermatology and conventional paper referrals.	Dahlén Gyllencreutz et al [42]
Randomized controlled trial	Single-center study in Australia	234 participants	Mobile teledermoscopy did not increase sensitivity for skin cancer detection in self-examination.	Janda et al [43]

^a^N/A: not applicable.

### Image Quality

In total, 8 papers about image quality in store-and-forward teledermatology were identified. The results are outlined in Table 3.

**Table 3:** Studies reporting image quality of teledermatology.

Type	Setting	Sample	Outcome	Reference
Observational	Single-center study in Austria	955 lesions	851 out of 962 (88%) dermoscopic lesions and 95 out of 123 (77%) clinical images noted to be of excellent quality.	Massone et al [13]
Observational	Single-center study in Denmark	519 patients	Substantial agreement noted between 2 dermatologists reviewing 600 images (AC2=0.68).	Vestergaard et al [18]
Observational	Single-center study in Austria	113 lesions	7% of images deemed inadequate for diagnosis.	Kroemer et al [20]
Observational	Single-center study in the Netherlands	108 teledermatoscopy referrals	The image quality was reported as bad in 36% of cases, reasonable in 28%, and good in 36%. Accuracy was improved in cases with good-quality images.	van der Heijden et al [23]
Randomized controlled trial	Single-center study in Switzerland	981 lesions from 39 general practitioners	2 (0.2%) images were deemed inadequate for inclusion.	Tandjung et al [36]
Observational	Single-center study in Brazil	333 lesions	12 cases (8.05%) were deemed inadequate. The introduction of a structured protocol increased the odds of acceptable imaging 38.77 times.	Piccoli et al [44]
Interventional	Single-center study in Sweden	172 lesions	Images were of intermediate to high quality in 95.5%-97.7% of primary care images and 96.5%-98.8% of dermatology images.	Dahlén Gyllencreutz et al [27]
Interventional	Single-center study in Australia	10 participants and 66 images	88% of images were deemed to be of good quality.	Janda et al [45]

## Discussion

### Principal Findings

The majority of studies assessing store-and-forward teledermatology are observational studies. Of the small number of interventional studies identified, only 2 were randomized controlled trials. The area with the most evidence available is for effectiveness, with 34 out of 45 studies reporting various outcomes of efficacy. Overall, the majority of studies in this literature review report that store-and-forward teledermatology services allow reduced time to advice, comparable effectiveness to in-person assessment, and at least adequate image quality for most skin lesions.

### Time to Advice and Management

One of the main advantages of teledermatology in skin lesion analysis is the potential for expedited treatment through the reduction of time between referrals and management. There have been several studies assessing this question. They predominantly show improvement with reduction ranging between 4 and 70 days for outcomes such as biopsy, definitive treatment, or clinic appointment [1-6]. Moreno-Ramírez and Ferrándiz [7] report reviewed 43,677 patients over 10 years with an average time to advice of 72 hours. Only 2 studies showed no difference in time to biopsy or definitive treatment [8,9]. A study by Duong et al [8] was underpowered with only 36 patients in the teledermatology pathway and compared in-person consults from 3 to 5 years earlier when there was potentially lower service demand. Very little detail on the teledermatology process used by Creighton-Smith et al [9] is provided. Perhaps, the lack of time reduction is a product of their teledermatology pathway design in this single-center study.

A retrospective review of 4589 referrals by Lowe et al [10] showed that using community imaging for skin lesion referrals enabled 86.3% of referrals to be managed without subsequent in-person assessment. A systematic review of 21 studies by Finnane et al [11] concluded that teledermatology could allow reduced wait times and earlier skin lesion management based on variable reduction in waiting time in 3 studies. Overall, there is a significant time-saving benefit when using teledermatology to review skin lesions.

### Effectiveness

The effectiveness of teledermatology is difficult to evaluate. One of the most prominent reasons is the heterogeneity in teledermatology services, making direct comparisons difficult. There is variable use of teledermoscopy and the provider experience in teledermoscopy. The study designs are often small, single-center studies, and the nature of teledermatology means that significant selection bias is often at play. The existing interventional studies often limit generalizability to clinical use by recruiting from settings other than primary care and have no way of allowing blinding. Another issue is the gold standard in diagnosis. Ideally, this would be a histological diagnosis; however, this is usually only available in a small proportion of the most suspicious lesions. In the absence of histology, often the reference standard defaults to in-person dermatologist assessment, which biases the diagnostic effectiveness away from teledermatology assessment. Despite this, a Cochrane review by Chuchu et al [12] focusing on diagnostic accuracy in teledermatology for skin lesion assessment concluded that teledermatology was accurate enough to diagnose most malignant lesions. This was a review of 22 studies published up to August 2016. Their estimate of the overall sensitivity was 94.9% (95% CI 90.1-97.4), and specificity 84.3% (95% CI 48.5-96.8) [12]. Even with their concerns regarding the quality of the studies, they recommended that teledermatology be used to triage patients requiring in-person assessment.

Overall diagnostic accuracy of teledermatology in assessing skin lesions is high. Multiple studies have shown it to be similar to in-person reviews, with figures ranging from 79.5% to 100% [13-17]. Often, this consists of high sensitivity and relatively lower specificity [18-21]. One study, focusing on 321 nonpigmented lesions for consideration of Mohs micrographic surgery, found that despite the high sensitivity of 92%, the specificity was only 49% [22]. van der Heijden et al [23] reported a lower accuracy, with only moderate teledermatology and histology agreement on 108 lesions, likely due to a high proportion of poor-quality images. The trend of high accuracy is consistent with a systematic review published by Finnane et al [11], which included studies from 2009 to 2015. This showed high diagnostic accuracy with the use of teledermatology. It was slightly inferior to in-person assessment at 51%-85% compared to 67%-85% [11]. The generally higher values of accuracy and sensitivity seen in studies from more recent years may reflect improvements in technology and greater experience with teledermatology diagnosis. Two studies looked specifically at positive predictive value when diagnosing melanoma [24,25]. They obtained values of 38.1% and 13.7%. The difference may reflect variability in dermatologist assessment with 9 readers, and significant variability within these noted in the latter study.

Similar to accuracy, there is a high degree of variation in diagnostic concordance reported in skin lesion teledermatology research. Overall, most studies indicate a high diagnostic concordance level compared to in-person dermatologist reviews and histopathology. A well-designed, repeated measures study by Warshaw et al [26] compared dermatologist and teledermatologist diagnoses of 3021 skin lesions from primary care. They report fair to substantial agreement for primary diagnosis of 45.7%-80.1% (κ=0.32-0.62) and substantial to perfect agreement for aggregated diagnoses (a primary diagnosis and up to 2 differential diagnoses) of 78.6%-93.9% (κ=0.77-0.9) [26]. Creighton-Smith et al [9] showed improved rates of partial concordance, and no difference in perfect concordance for teledermatology diagnoses compared to in-person dermatology diagnoses at 52% versus 36.3% (*P*<.05) and 36% versus 43.1% (*P*=.4), respectively. Dahlén Gyllencreutz et al [27] reported high interobserver agreement in skin lesion diagnosis between 2 dermatologists reviewing 172 images from primary care (81.4% and 83.7%). Zink et al [28], Arzberger et al [29], Lamel et al [30], Carter et al [5], and Kroemer et al [20] reported high diagnostic concordance for teledermatology compared to in-person or histology. In contrast, a small study of 10 lesions showed higher concordance for in-person review than teledermatology (κ=0.6 vs 0.52) [31]. Greenwald et al [32] conducted a real-world study assessing the histological diagnosis of pigmented lesions excised on the advice of a store-and-forward teledermatology service. In total, 260 of 1572 lesions (17%) were found to be melanoma, leading to a benign:malignant ratio of 4.9:1 [32]. This reduced with increased dermatologist diagnostic confidence to 2.8:1 for “excise, possible melanoma” and 1:1 for “excise, likely melanoma” when compared to the default “excise to remove doubt.”

A benefit of teledermatology is the reduction in patients requiring in-person assessment. A retrospective review of 59,279 primary care referrals found a 39% decrease in face-to-face appointments using the teledermatology pathway [33]. Mehrtens et al [34] found that teledermatology allowed 50% of referrals to be discharged to GPs and 33% to proceed straight to biopsy, saving an estimated 16,282 in-person appointments. Several other studies also noted substantial reductions in face-to-face appointments after implementing teledermatology services [13,35,36].

One of the most important distinctions when reviewing skin lesion assessment by teledermatology is the use of teledermoscopy. This is particularly important when pigmented lesions are assessed. Most research suggests adding dermoscopy images for teledermatology is more effective than clinical images alone. Reviews by Beer et al [37] and Woodley [38] concluded that teledermoscopy increases lesion diagnosis accuracy. Gómez Arias et al [39] compared diagnostic concordance with and without teledermoscopy in 395 cases and showed increased concordance when dermoscopy was included. Şenel et al [40] reported improved accuracy in diagnosing 150 patients with nonpigmented lesions with dermoscopy compared to without (0.86 and 0.88 vs 0.75 and 0.77; *P*<.05 for 2 different dermatologists). Several studies have reported the high effectiveness of teledermatology when using dermoscopic images [24,41,42].

In contrast, Janda et al [43] and de Giorgi et al [31] found no increase when dermoscopy images were added to teledermatology assessments. In the former, images were acquired directly from 234 participants through skin self-examination. Images were likely to be particularly poor if participants were unaware of the need to optimize the skin surface before obtaining dermoscopy imaging. The latter was a small trial of 10 lesions, and as the authors note, there were many difficult lesions.

The availability of clinical history is another factor that may impact teledermatology diagnosis. Surprisingly, it is difficult to find details on this area within studies, making the effect of this difficult to determine.

### Image Quality

Image quality is an essential aspect of skin lesion teledermatology. Many studies have reported the proportion of images adequate for diagnosis. These range from 0.2% to 36% [13,18,20,23,36,45]. Dahlén Gyllencreutz et al [27] compared image quality from primary care practices using iPhone 4 cameras with dermoscopy attachments to those taken by dermatologists with Canon EOS D550 cameras. In total, 172 cases were reviewed by 2 dermatologists not familiar with the cases. Despite the differences in camera resolution and operator experience with dermoscopy, there was no statistical difference in the quality of images obtained. The evaluators rated 1.7% and 4.7% of primary care images as poor compared to 1.2% and 3.5% of dermatology images (*P*=.25 and *P*=.28) [27]. They postulate that the difference between evaluators reflects the subjective nature of image quality assessment. Vestergaard et al [18] also had individual dermatologists review the quality of 600 images from primary care. Unlike Dahlén Gyllencreutz et al [27], Vestergard et al [18] found substantial agreement in image quality, with approximately 10% reported as poor quality by each reviewer.

Image quality in teledermatology depends on several factors, such as the equipment used, the inclusion of dermoscopy, and the experience of the person taking images. Studies have shown images obtained by dermatologists or dermatology nurses to be of higher quality than those from primary care [23]. Images obtained by patients generally have the lowest image quality [45]. This is likely due to the increased difficulty taking the images, less experience in focusing lesions, and in the case of dermoscopy, unfamiliarity with the need to prepare the skin, such as removing make-up or applying a fluid to the lesion. Given advances in imaging quality, notably improved smartphone camera resolution, more recent research is likely to report higher image quality regardless of the operator.

### Conclusions

Overall, teledermatology offers a comparable standard of effectiveness to in-person assessment. It can save significant time in expediting advice and management. Image quality and inclusion of dermoscopy have a considerable bearing on the overall effectiveness. There is a need for large interventional studies, particularly those with high proportions of histology available to enable definitive conclusions regarding teledermatology outcomes. There is a gap in the literature for studies comparing different teledermatology methods.

## Abbreviations

GP: general practitioner
